# Spurious History

**Published:** 1895-12

**Authors:** 


### Editorial.

### SPURIOUS HISTORY.

The readers of journals must have been impressed with the fact that very little attention is ordinarily paid to historical verification of statements made. So serious has this careless handling of subjects become that it is as much of an evil as the plagiarism which we have had occasion to allude to in a former number, and which has been a blot upon the literary work of the profession for years.

Writers seem to forget that a statement involving an historical fact should never be made until all possible means of verifying its accuracy have been exhausted. The responsibility is a very great one. The difficulties surrounding this effort are many and not easily overcome, for it means a research into the very origin of things pertaining to dentistry, and a thorough acquaintance with its literature and that of collateral subjects not possessed by the many, and, probably, in its entirety not by any one.

It not infrequently happens that writers will quote the last article read as authority without the slightest effort to discover its truthfulness, when, if the facts were known, it would be made clear that the supposed original writer had thoughtlessly or criminally assumed that his statement ought to have been correct, and affirmed it accordingly. The next writer, feeling the obligation to quote, refers to this or copies without credit, and thus the false statement goes ringing down the years until a later generation accepts it as a fact.

One of the most notable examples of this in recent years has been seen in the various papers and discussions upon pyorrhœa alveolaris. The attempts made to give correct history on this subject would be amusing were it not so serious. It would be difficult to give the countless blunders made in the history of this subject, errors which it will require years to rectify. We have tried many times to enforce the truth of history in regard to this, that we are indebted to the older, as well as the modern, French writers for the first and most thorough descriptions of this pathological condition. It was with more than ordinary satisfaction that we welcomed in our last number Dr. C. N. Peirce's timely work in his historical *résumé* of the labor performed by the French dentists upon this subject, and it is to be hoped that this paper will settle for all time the historical side of the question, and relegate the former crudities of statement to oblivion.

This, however, is only typical of a whole series of errors ranging from the absolutely false to the supposed discovery of *new things and new methods* already hoary with age.

Perhaps one of the worst features of this mutilation of history is the attempt, frequently made, to belittle the work of the past. If some writers could be believed, there was no such thing as a good filling made fifty years ago; that the practitioners of that period had no knowledge of mechanical dentistry or methods of treatment; in fact, in the light of present superior knowledge, are not worthy the slightest recognition. Indeed, if these writers are to be believed, the work of the fathers was but a crude basis for the more perfect superstructure of which they form a part in the present. The men of the middle half of the nineteenth century are fast passing away, and soon there will be none left to bear personal witness to the truth of the assertion, which we unhesitatingly make, that the mechanical and operative work was, at that period, as effective as at the present; and the mechanical work of the past decade can claim no superiority over that of fifty years ago; indeed, it is doubtful whether it can compare with it in character and durability. It is to be questioned whether any of the younger generation of workers have ever seen such exquisite pieces of plate work as were turned out by Reynolds, originally of Geneva, N. Y. Such a thing as his double-backed gold plates are unknown, as far as we are aware, at the present time.

It was, therefore, with something of a shock that we read in the September number of the *Dental Review* the following paragraph, part of the discussion upon a question on "Operative Dentistry":

"There is no gentleman present this evening who has entered into anything like a retrospect of the injury that was done to the manipulative ability of the dentist during the period from 1850 to 1880 by the introduction of vulcanite and the introduction of nitrous oxide gas for the extraction of teeth. I began the study of dentistry in 1867, and my preceptors began the study of it in 1855, *and neither of them could make a gold plate, because they had not been taught to do it.* There was *scarcely any instruction in any of the dental colleges between 1851 and 1852 and 1865 with reference to the manufacture and fabrication of metal plates, and the demonstrator himself,* as a rule, *was not capable of doing it. Consequently, the whole body of the dental profession nearly were unable to do metal plate work.* The ease with which teeth were extracted *through the administration of nitrous oxide,* and the *ease with which artificial teeth were inserted,* decreased the value of the services of dentists all over the country. If you went into little towns at that time *you would rarely find a man who could put in gold fillings.* He did not dare to do it, *because cement fillings were being inserted for fifty cents a piece.*"—Harlan (italics ours).

It seems incredible that any one should have made such statements, and particularly one occupying the position of the speaker. We should have been inclined to believe that it was a reportorial error had the paragraph not had editorial supervision.

We would beg leave to call the speaker's attention to the fact that up to 1850, and for some time thereafter, nothing but gold and silver were used as bases for artificial teeth in this country. Ivory and bone were used in Europe, as well as the metals. The "moulded" or poured tin base was made by Hudson, of Philadelphia, about 1820; but it was not used, to any extent, until Dr. George E. Hawes, in 1850, revived the process, and even then it was not generally adopted. It is in the last degree absurd to suppose that men who had nothing else to depend upon but gold and silver should not have become skilled in its use. It will be news to those now living who practised in the period alluded to, to be told that "the ease with which artificial teeth were inserted decreased the value" of their services; and it is still more singular that the extraction should have been accomplished "through the administration of nitrous oxide," inasmuch as this agent was not brought into general dental practice, as an anæsthetic, until introduced by G. Q. Colton in 1863, although originally applied by Wells in 1844.

It was not until 1855 that a patent was taken out in England, by Goodyear, for making a dental plate of hard rubber, and in this country by Cummings in 1855; but its use was by no means general, even as late as 1860. The first denture inserted by the writer was in 1858. The influence of vulcanite was, therefore, not marked in the decade between 1850 and 1860.

It is sad to think of what little value the teachings of Maynard, Harris, Townsend, Arthur, Westcott, Dwinelle, Rich, and a host of others could have been, that you could "rarely find" in the "little towns a man who could put in gold fillings." It was in one of the little towns in 1848 that the writer met a man who could place in gold equal to a Varney or a Webb. The reason given that the dentist "did not dare to do it because cement fillings were being inserted for fifty cents," seems rather out of place, in view of the fact that the first cement introduced, of any value, was the oxychloride of zinc, the invention of M. Sorel, in 1856, for stucco work, but did not come into dental use for several years thereafter.

The dentist of these earlier decades was forced to make his work with a reasonable degree of thoroughness, and all dentists worthy the name were instructed in the manipulations in private offices and in colleges. To say that nothing of this kind was taught in the latter is a libel on these institutions. That this may not have been done with the care with which it is attempted to-day is possible, for the very excellent reason that students were all, with few exceptions, familiar with the work before entering the schools, and not, as now, taken without previous preparation.

We do not know the college from whence the preceptors of the speaker received their diplomas. They certainly were not of the kind familiar to the writer, if the demonstrators were unable to make a metal plate. Such a serious lack of knowledge would have ended in an early dismissal from the service.

The object of this article is not to criticise the historical failings of individuals, but rather to use these as an example to impress the fact generally that accuracy of statement was never more needed in speaking and writing than to-day in dentistry. It must be remembered that this profession is yet in its youth, and it is vitally important that the details of experience be correctly gathered as we advance to a broader and more exact knowledge.

